# Anodal Transcranial Direct Current Stimulation Reduces Psychophysically Measured Surround Suppression in the Human Visual Cortex

**DOI:** 10.1371/journal.pone.0036220

**Published:** 2012-05-01

**Authors:** Daniel P. Spiegel, Bruce C. Hansen, Winston D. Byblow, Benjamin Thompson

**Affiliations:** 1 Department of Optometry and Vision Science, University of Auckland, Auckland, New Zealand; 2 Department of Psychology & Neuroscience Program, Colgate University, Hamilton, New York, United States of America; 3 Department of Sport and Exercise Science, University of Auckland, Auckland, New Zealand; University of Leuven, Belgium

## Abstract

Transcranial direct current stimulation (tDCS) is a safe, non-invasive technique for transiently modulating the balance of excitation and inhibition within the human brain. It has been reported that anodal tDCS can reduce both GABA mediated inhibition and GABA concentration within the human motor cortex. As GABA mediated inhibition is thought to be a key modulator of plasticity within the adult brain, these findings have broad implications for the future use of tDCS. It is important, therefore, to establish whether tDCS can exert similar effects within non-motor brain areas. The aim of this study was to assess whether anodal tDCS could reduce inhibitory interactions within the human visual cortex. Psychophysical measures of surround suppression were used as an index of inhibition within V1. Overlay suppression, which is thought to originate within the lateral geniculate nucleus (LGN), was also measured as a control. Anodal stimulation of the occipital poles significantly reduced psychophysical surround suppression, but had no effect on overlay suppression. This effect was specific to anodal stimulation as cathodal stimulation had no effect on either measure. These psychophysical results provide the first evidence for tDCS-induced reductions of intracortical inhibition within the human visual cortex.

## Introduction

Transcranial direct current stimulation (tDCS) is becoming widely used as a technique for non-invasively manipulating excitability in the human cortex [1], [2], [3], [4], [5]. During tDCS, a weak direct current is delivered to a specific cortical region using two electrodes placed on the scalp. The stimulation can result in a polarity specific change in neural excitability, whereby cathodal tDCS tends to reduce excitability and anodal tDCS tends to increase excitability [1], [4]. In addition, it has recently been demonstrated that anodal tDCS can reduce intracortical inhibition [6], [7], possibly by lowering GABA concentration [8]. The implications of this property of tDCS are significant as abnormal inhibitory interactions have been implicated in a number of neurological disorders such as Rett syndrome, Down syndrome, autism, schizophrenia and amblyopia [9], [10]. Furthermore GABA mediated inhibition has been identified as a key factor in gating plasticity in the adult brain and in the adult visual cortex in particular [11], [12], [13], [14], [15]. Based on previous work focusing on the human motor cortex [6], [7], [8], [16], this study was designed to test the hypothesis that anodal tDCS could reduce GABA mediated inhibition within the human primary visual cortex.

There are two distinct suppressive neurophysiological mechanisms within the visual system that can reduce the response of a cell to a stimulus when a “target” stimulus is presented in conjunction with a “mask”. These two types of suppression are distinguished by the position of the mask with respect to the neuron's receptive field. Surround suppression occurs when the mask surrounds the target [17], [18] and overlay suppression (also known as cross-orientation masking) occurs when the mask is superimposed upon the target [19], [20], [21]. Analogues of these effects can be measured psychophysically, whereby the contrast detection threshold for a target stimulus is increased in the presence of a surround or overlay mask [21].

Surround suppression is thought to originate within the primary visual cortex [22], [23], [24] and recent studies strongly imply that surround suppression involves GABA mediated inhibition [25], [26], [27]. For example, it has been demonstrated that psychophysical measures of surround suppression are correlated with GABA concentration within the visual cortex [25].

Conversely, neurophysiological studies in the cat suggest that overlay suppression depends on LGN inputs to V1 [28], [29], [30], [31] and that this type of suppression is not reliant upon GABA mediated inhibition within the visual cortex [32]. This is in agreement with the findings of Petrov et al. [21] who demonstrated psychophysically that overlay suppression precedes surround suppression within the human visual pathway. Specifically, it was shown that an orthogonal overlay mask superimposed on the surround mask, reduced the inhibitory effects of the surround mask on target detection. In other words, the surround mask was suppressed by the overlay mask. Conversely, the addition of a surround mask did not influence the inhibitory effect of an overlay mask on target detection [21].

Previous studies have demonstrated that tDCS can influence neural activity within the human visual cortex [33], [34]. For example, Antal at al. [3] reported a decrease in phosphene threshold (increased excitability) following anodal tDCS and an increase in phosphene threshold (reduced excitability) following cathodal tDCS of the primary visual cortex. tDCS of the primary visual cortex has also been shown to influence visual evoked potentials (VEPs) whereby anodal stimulation increased and cathodal stimulation decreased the amplitude of the N70 VEP component [35]. Opposite results have been reported for the P100 component of the VEP [36]. It has also been demonstrated that tDCS of the visual cortex can influence visual perception. In a recent study, Kraft and colleagues [37] reported a polarity-specific change in contrast sensitivity measured using threshold perimetry. Anodal tDCS increased contrast sensitivity within the central 2° of the visual field whereas cathodal tDCS had no effect. Conversely Antal et al. [38] found no improvement in contrast sensitivity after anodal tDCS but a decrease in sensitivity after cathodal tDCS.

To assess whether anodal tDCS could reduce inhibitory interactions within the human visual cortex, we measured the effect of anodal primary visual cortex tDCS on psychophysical measures of surround suppression. To control for any general effects of tDCS we also applied cathodal stimulation to the visual cortex, as cathodal stimulation is not thought to reduce inhibition within the stimulated brain area [8]. As an additional control we also assessed the effects of both anodal and cathodal stimulation on overlay suppression. Since overlay suppression is thought to originate within the LGN and does not appear to recruit GABA mediated inhibitory networks, we did not anticipate a measureable effect of tDCS on this type of suppression.

## Materials and Methods

### Ethics Statement

This study was approved by the Northern X Regional Ethics Committee, New Zealand and all study protocols were in accordance with the Declaration of Helsinki. All participants gave full written informed consent prior to taking part in the study.

### Subjects

Eleven healthy participants (4 females) aged between 23 and 32 years (mean 28.9 years) gave written informed consent. All participants completed baseline psychophysical measurements; however, one participant did not show evidence of surround or overlay suppression and was excluded from the study. Therefore data for 10 participants are reported below. All of the participants had normal or corrected to normal vision and wore their habitual optical correction during testing.

### Stimuli

**Figure 1:**
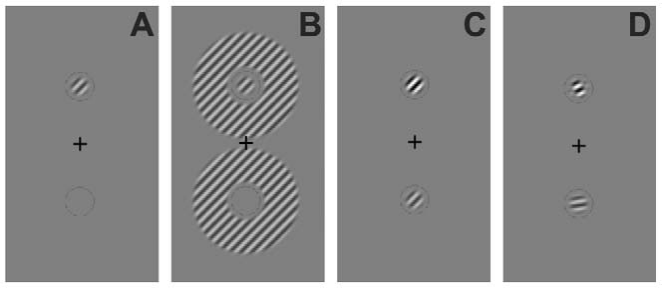
Psychophysical stimuli. The target is located above the fixation cross in all panels. Panel A. shows the no mask condition for which only the target was shown. Panel B shows the surround masking condition (mask orientation offset 0°). Panel C shows the overlay mask condition with a mask orientation offset of 0° (i.e. the mask and target are collinear). Under these conditions the mask and target contrast is summed and target detection is facilitated. Panel D shows the overlay mask condition with a mask orientation offset of 35° which suppresses the target (increases the detection threshold). Note that the targets are shown at high contrast in this figure for illustrative purposes.

The stimuli were based directly on those used in previous psychophysical studies of visual masking [21], [39] and are shown in figure 1. The target stimulus was an obliquely oriented (45°) Gabor patch (a sinusoidal luminance pattern presented within a Gaussian envelope) subtending 0.45° of visual angle with a spatial frequency of 3.5 cpd and a variable contrast. The target stimulus was presented either above or below fixation at an eccentricity of 1.2°. This eccentricity was chosen for two reasons. Firstly, it has previously been demonstrated that surround suppression cannot be measured in the fovea using a psychophysical paradigm involving contrast thresholds [21]. Secondly, the effect of tDCS has been shown to be less pronounced in the peripheral visual field [37], [40], presumably due to the retinotopic organization of the calcarine sulcus, whereby the central visual field is represented more superficially than the periphery [41]. Our chosen eccentricity therefore provided a suitable compromise between these two factors. In order to reduce uncertainty regarding the location of the target, the two potential target locations were indicated by thin low contrast circles [21] (figure 1). The surround mask consisted of a 2.18° diameter annulus constructed from a sinusoidal grating of 40% Michelson contrast defined as 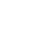, where Ia = maximum luminance and Ib = minimum luminance, and with the same spatial frequency and phase as the target. The inner edge of the annulus was located 0.37° from the centre of the target. The overlay mask consisted of a Gabor patch of 20% Michelson contrast identical to the target stimulus in size, spatial frequency and phase. The stimuli were presented on a uniform grey background (luminance 27 cd/m^2^).

To determine the contrast detection threshold of the target in both masked and unmasked conditions, we employed a two-alternative forced choice (2AFC) paradigm in which the target appeared either above or below the fixation cross and a standard 1-up-2-down adaptive staircase procedure [42] in which the target contrast was varied using a step size of 0.5% Michelson contrast over 12 staircase reversals. The starting contrast was 20%. Although eye movements were not recorded as part of this study, participants were thoroughly trained on the task to ensure that they were able to maintain stable fixation and make perceptual judgements in the near periphery. In addition, the stimuli were presented for 150 ms, a duration short enough to prevent eye movements [43], [44].

The self-paced task was to report whether the target was presented above or below the fixation cross. The detection threshold was calculated as the average of the last five reversals of the staircase. Each threshold was measured 4 times per session. In the masking conditions, identical masks were presented both above and below the fixation cross. Following Petrov et al. [21], the suppressive effect of the masks on contrast detection thresholds for the target was quantified as a suppression factor, a well-established measure of visual masking [31], [45]. The suppression factor was defined as the contrast detection threshold of the masked target divided by the contrast threshold of the target alone. If the presence of a mask increased the target detection threshold, the suppression factor was greater than 1, whereas a suppression factor less than 1 implied mask-induced facilitation. The four threshold measurements for each condition were averaged prior to the calculation of the suppression factor.

The stimuli were generated using PsychToolBox version 3.0.8 for MatLab installed on Mac Mini 2 GHz Intel Core 2 Duo, 4 GB 1067 MHz DDR3 and viewed on a Sony CPD G520 CRT screen with resolution of 1600×1200 pixels and a refresh rate of 85 Hz. The monitor was linearly calibrated using a Minolta LS-106 luminance meter, and a bit–stealing algorithm [46], [47] was used to yield 10.8 bits of luminance resolution. Participants were seated in a comfortable chair and viewed the display screen from a distance of 1 meter. As it has been shown that binocular viewing may protect against the effects of non-invasive brain stimulation on a visual perceptual task [48], possibly by providing a more robust cortical representation of the visual stimuli [49], participants viewed the stimuli monocularly with an opaque patch over their non-dominant eye.

### Procedure

**Figure 2:**
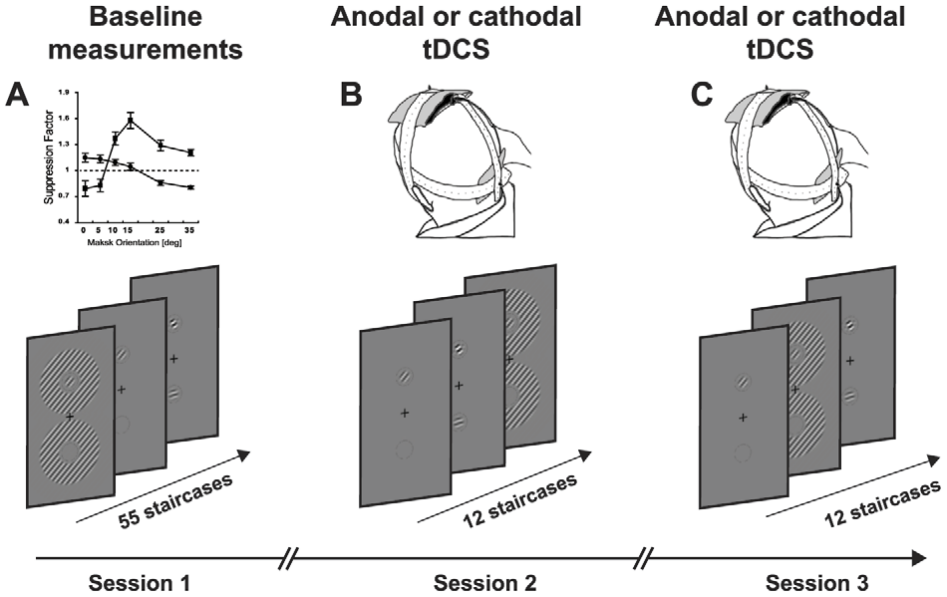
Experimental design. The study had two phases. Firstly baseline measurements of both types of suppression were quantified across a range of mask orientations (A). One orientation that produced measureable suppression was then chosen for each type of mask for psychophysical measurements made during anodal and cathodal tDCS (B and C). The sequence of stimulus presentation was randomized within each session.

The study had two phases to allow for psychophysical measurements of overlay and surround suppression to be conducted within a timeframe suitable for tDCS stimulation (figure 2). Firstly, each participant completed a set of baseline measurements where contrast detection thresholds for the target stimulus were assessed across a range of overlay and surround mask orientations. A mask orientation that produced measureable suppression was then selected for each individual participant for each of the two mask types (overlay and surround). This resulted in a set of three stimuli for the measurements made during tDCS stimulation: 1) the no mask condition, 2) an overlay mask condition with the mask fixed at a chosen orientation and 3) a surround mask condition with the mask fixed at a chosen orientation. These stages are described in detail below.

### Baseline measurements

Two baseline measurement sessions were conducted. The first was conducted to ensure that the participants were familiar with task and the second provided threshold estimates that were used in the second stage of the study. Each baseline measurement session consisted of contrast detection measurements for the target alone, the target with an overlay mask and the target with a surround mask. Both the overlay and surround masks were presented at six different orientation offsets from the target orientation; 0° (collinear with the target), 5°, 10°, 15°, 25° and 35°. The conditions were measured in a randomised sequence and the threshold for each condition was measured 4 times. Participants were advised of the upcoming condition by a text prompt presented on the stimulus display screen prior each staircase. In addition, three staircases (one target alone, one with an overlay mask and one with a surround mask) were measured at the start of each session to provide practise trials. This gave a total of 55 staircase measurements per baseline session, 4 staircases for the target only condition, 48 staircases for the target plus mask conditions (2 masks ×6 orientations ×4 staircases = 48) and three practise staircases. Each session was split into two blocks to avoid fatigue.

### Selection of mask orientations for the use during tDCS

One overlay mask orientation and one surround mask orientation were chosen from the baseline measurements for use during tDCS on an individual participant basis. Mask orientations were selected with the aim of having as a little variation as possible across the chosen orientations. An inspection of the baseline data for all participants revealed that 5/10 participants showed reliable suppression (defined as a suppression factor greater than 1.1) at an orientation offset of 10° for both mask types. Therefore a 10° mask orientation offset was chosen for this group for both masks. For the remaining five participants we selected the surround mask orientation offset that resulted in the strongest suppression and the closest overlay mask orientation offset that resulted in the same or higher level of suppression. Individual mask orientations and baseline suppression factors for both masking conditions are provided in table 1.

**Table 1:** Individual mask orientations and baseline suppression factors for both masking conditions.

Subject no.	Surround Mask Offset [deg]	Surround Mask Suppression Factor	Overlay Mask Offset [deg]	Overlay Mask Suppression Factor
**1**	5	1.13	15	1.59
**2**	10	1.19	10	1.62
**3**	0	1.22	15	1.72
**4**	10	1.12	10	1.67
**5**	0	1.16	15	1.43
**6**	15	1.09*	15	1.33
**7**	10	1.28	10	1.42
**8**	0	1.22	10	1.51
**9**	10	1.18	10	1.78
**10**	10	1.11	10	1.44
**Mean**	7	1.17	12	1.55
**SEM**	1.70	0.02	0.82	0.05

The asterisk indicates maximum surround suppression factor of a subject 6 who did not show a suppression factor of 1.1 at any orientation.

### Measurements made during tDCS

To assess anodal tDCS-induced effects on overlay and surround masking, all participants attended two sessions each consisting of 12 randomly sequenced staircases completed during tDCS of the visual cortex. Anodal stimulation was delivered to the visual cortex in one session and cathodal stimulation, which is not thought to reduce inhibitory interactions within the human cortex, was delivered in the other session as an active control. The order of these sessions was randomized and the sessions were separated by at least two days. The contrast detection threshold for the target stimulus was measured 4 times for each condition (no mask, overlay mask and surround mask) in a randomized sequence. The orientations of the surround and overlay masks were selected from the baseline psychophysical measurements on an individual participant basis as described above. Baseline measurements were not repeated directly prior to tDCS administration to avoid fatigue and any associated variability in the psychophysical data. However, the behavioural task was designed to account for this. All three stimulus configurations were measured during each tDCS session (target-alone, target + surround and target + overlay). As the suppression factor for a particular test session was calculated with reference to the target-alone threshold measured during that specific session, the data were robust to factors affecting general task performance. In addition, any between session variability would have been equally distributed across the anodal and cathodal (control) stimulation sessions. The minimal interval between the baseline and measurements and the first tDCS session was 2 hours and the mean was 17 days (SEM = 4). When there was an interval longer than 14 days between any two sessions, a subject completed a minimum of 22 staircases in order to re-familiarize them with the task and ensure that baselines were stable.

### Transcranial direct current stimulation

tDCS was generated using a 9 V battery driven direct current stimulator (Chattanooga Ionto, USA) and delivered via a pair of rubber electrodes (Speds Medica S.r.l., Italy) covered in saline-soaked sponges. The size of the stimulating electrode was 72×60 mm and the size of the reference electrode was 115×95 mm, rendering the large reference electrode inert due to low current density [50]. The positioning of the electrodes was adopted from previous tDCS studies related to vision [3], [4], [33],[34],[35],[38] whereby the stimulation and reference electrode were centred over Oz and Cz respectively, in accordance to the international 10-10 EEG system [51]. It has been suggested that tDCS has shorter lasting aftereffects for the visual cortex than for other cortical areas and it is known that the aftereffects decay over time [3], [52]. As the suppression factor calculation used in this study required comparisons between measurements that were made over the course of several minutes, we chose to perform the psychophysical measurements during stimulation rather than after stimulation. This was because we assumed that the effects of tDCS would be more stable over time during stimulation than after stimulation.

For both anodal and cathodal stimulation, the current was initially ramped up over 31 seconds to an intensity of 2 mA and then kept constant. In order to obtain the most accurate psychophysical data possible, we chose not to fix the stimulation duration. Rather, we fixed the number of self-paced staircase measurements completed by each participant at 12 and terminated stimulation as soon as these measurements were complete. The stimulation time therefore varied between 8 and 17 minutes and depended on the participant's response rate.

### Statistical analysis

A repeated measures ANOVA (degrees of freedom corrected for sphericity using the Huynh-Feldt correction where necessary) with factors of mask (surround vs. overlay) and mask orientation (0, 5, 10, 15, 25, 35°) was conducted on the baseline suppression factors to identify any differences in suppression relating to the use of overlay vs. surround masks prior to tDCS. Post-hoc paired two sample t-tests were then used to evaluate differences in suppression between the two mask types at each mask orientation offset.

In order to assess the effect of anodal and cathodal stimulation on each type of suppression, repeated measures ANOVAs with a factor of stimulation (no stimulation/baseline, anodal stimulation, cathodal stimulation) were conducted on the data for the surround mask and the overlay mask conditions separately. Post-hoc paired two sample t-tests were then used to compare pairs of stimulation conditions for each mask type separately. Repeated measure ANOVAs, also with a factor of stimulation, were conducted on the target alone threshold data to test for any effects of tDCS on detection thresholds in the absence of a mask. To further control for any tDCS induced changes in detection threshold for the target alone that may have influenced the suppression factor, an ANCOVA with a factor of stimulation (no stimulation/baseline, anodal stimulation, and cathodal stimulation) and a covariate of change in target stimulus threshold induced by anodal tDCS baseline threshold – threshold measured during anodal tDCS) was conducted.

## Results

**Figure 3:**
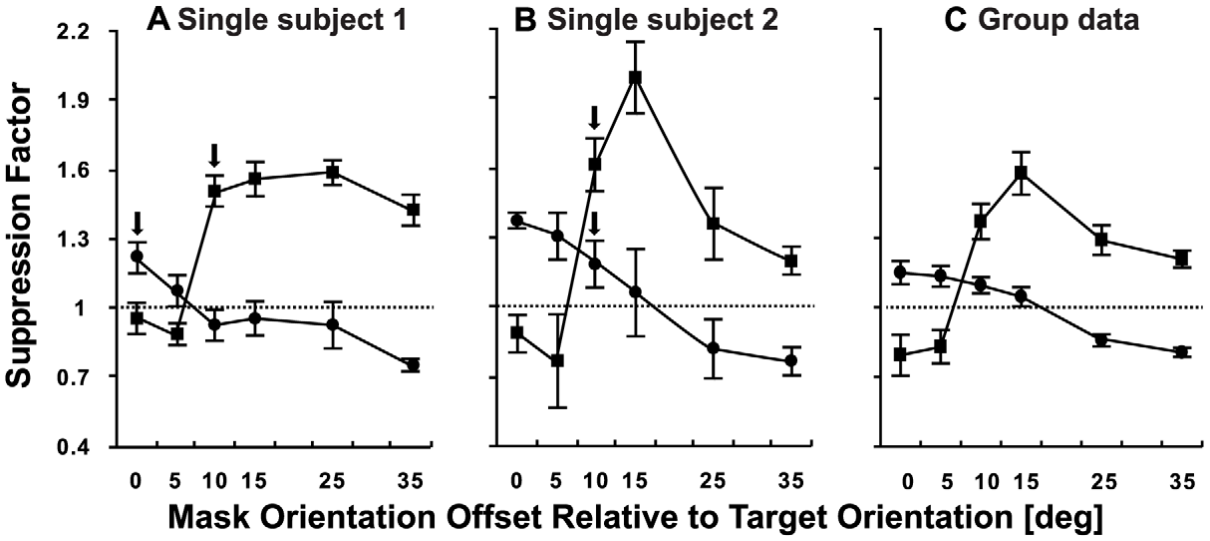
Psychophysical measurement of suppression for the surround (circles) and overlay masking (squares) conditions. Panels A and B show individual participant data and C shows the mean group data. The suppression factor shown on the y-axis of each panel was calculated by dividing the detection threshold for the target alone by the threshold for the target + mask. Values above 1 (dashed line) indicate mask-induced suppression. Arrows in A and B indicate the mask orientations used for the tDCS portion of the study. Error bars represent ±1 SEM.

Pre-tDCS measurements indicated that the stimulus configuration allowed for both surround and overlay suppression (figure 3) consistent with previous work [21]. An ANOVA with factors of mask (surround vs. overlay) and orientation (0, 5, 10, 15, 25, 35°) revealed significant main effects of mask (F_1, 9_ = 14.869, p<0.01) and orientation (F_5, 45_ = 12.568, p<0.001) and a significant interaction between these two factors (F_5, 45_ = 30.841, p<0.001). Post hoc paired t-tests revealed significant differences in the amount of suppression generated by each mask type for every mask orientation tested (p<0.01). The main effect of mask type was driven by significantly stronger suppression for the overlay mask at orientation offsets from the target of 10° or greater. The interaction effect was due to a gradual shift from suppression to facilitation with increasing mask orientation offset for the surround mask data and an abrupt change from facilitation to suppression for mask orientations >5° for the overlay mask (figure 3).

As described earlier, the choice of mask orientations for the tDCS portion of the study were based on each participant's psychophysical data. The average mask orientation used during tDCS was 7° (SEM = 1.70°) for the surround condition and 12° (SEM = 0.82°) for the overlay condition. Examples of individual psychophysical results are provided in figures 3A and 3B.

In order to assess the tDCS-induced effects on both types of suppression, suppression factors obtained during the baseline (no stimulation) measurements were compared with suppression factors measured during anodal and cathodal stimulation (figure 3) using a repeated measures ANOVA. An ANOVA conducted on the surround masking data revealed a main effect of stimulation indicating that tDCS influenced the suppression factor for this mask type (F_2, 18_ = 5.978, p = 0.01). This was not the case for the overlay masking condition however where no significant main effect of stimulation was apparent (F_2, 18_ = 0.505, p = 0.612).

For the surround mask condition both anodal and cathodal stimulation decreased the suppression factor; anodal by 14.8% (SEM = 4.8) and cathodal by 4.7% (SEM = 5.3). However, post-hoc two tailed paired sample t-tests indicated that while the effects of anodal tDCS differed significantly from baseline t_9_ = 3.231, p = 0.01, the effects of cathodal tDCS did not t_9_ = 0.899, p = 0.392. In addition, the effect of anodal stimulation was significantly different from the effect of cathodal stimulation t_9_ = −3.692, p<0.005.

**Figure 4:**
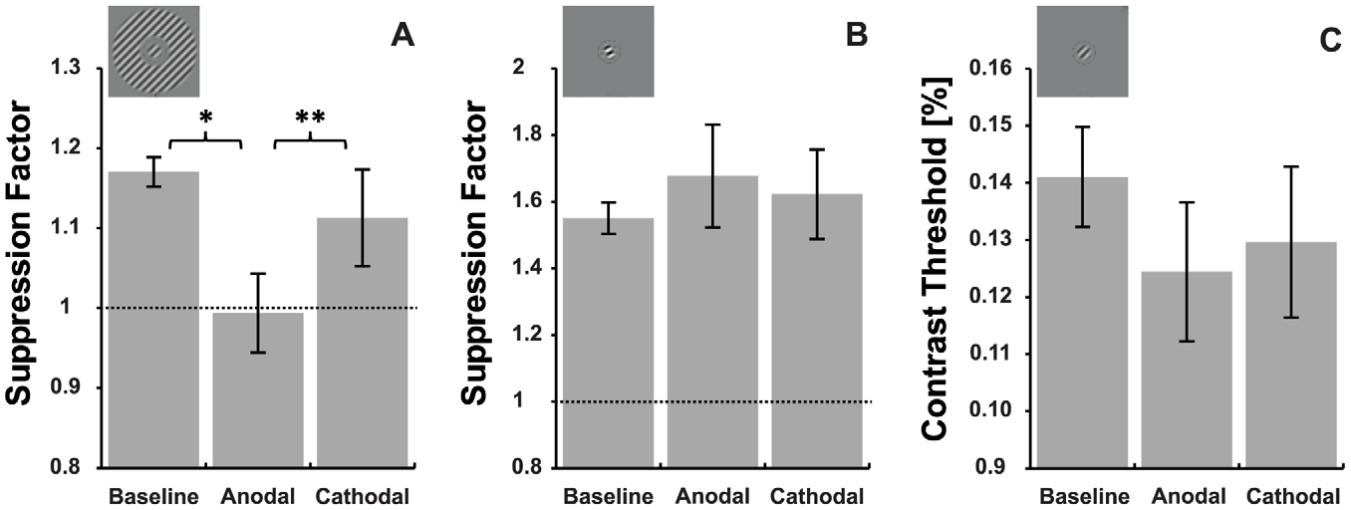
The effect of tDCS on surround and overlay masking. Panel A shows data for surround masking, panel B shows data for overlay masking. * p<0.05, ** p<0.01 (two tailed paired t-test). Dashed lines indicate no suppression. Panel C shows the mean contrast detection thresholds for the target with no mask in place. Error bars represent ±1 SEM. Note that error bars represent between subject error, whereas p values represent within-subject differences.

**Figure 5:**
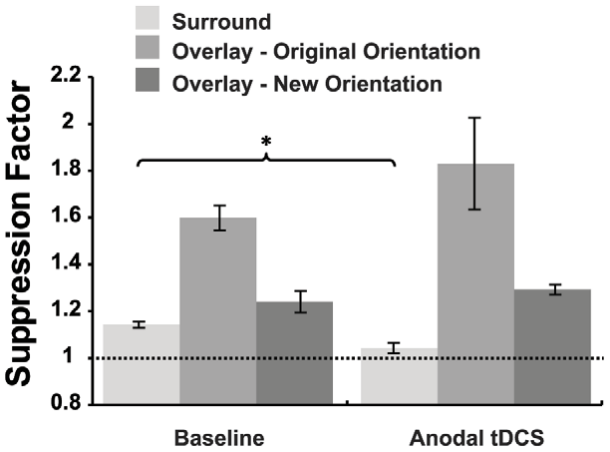
The effect of anodal tDCS on weaker overlay masking for a subset of 5 subjects. Data are shown for surround masking and the original overlay mask orientation as well as for the overlay orientation that generated the closest possible suppression factor to the surround suppression factor for a subset of 5 subjects. Dashed lines indicate no suppression. * p<0.05, (one tailed paired t-test) Error bars represent ±1 SEM.

As can be seen in figure 4, the choice of identical or similar mask orientations for the surround and overlay conditions resulted in significantly greater suppression for the overlay condition than the surround condition (t_9_ = −8.059, p<0.001). This raised the possibility that the lack of an anodal tDCS effect on overlay suppression was due to the greater levels of suppression in the overlay condition. An additional set of measurements were therefore conducted for a subset of 5 participants to test the effect of anodal tDCS on weaker levels of overlay suppression. For these participants an overlay mask orientation that gave the closest level of suppression to the surround mask orientation used in the main experiment was tested during anodal tDCS. Due to the rapid change from facilitation to strong suppression with increasing mask orientation offset that characterised the overlay mask data (figure 3C), the revised overlay mask orientations still generated larger suppression factors than the surround masks (mean difference 0.09, SEM = 0.05), however this difference was no longer statistically significant (one tailed paired t-test, t_4_ = 1.350, p = 0.124). In agreement with the main experiment, anodal tDCS did not induce a reliable change in overlay mask suppression which increased by an average of 4.2% (one tailed paired t-test, t_4_ = 0.639, p = 0.279) (figure 5). To further investigate the potential effect of suppression strength on tDCS effects, we measured the relationship between the baseline surround suppression factor and the suppression factor obtained during anodal stimulation for the data from the main experiment. This correlation was not significant r = −0.102, p = 0.779, suggesting that the amount of surround suppression present prior to stimulation did not predict the response to tDCS. These findings are in agreement with previous reports that the ratio between excitation and inhibition does not predict the responsiveness of the motor cortex to anodal tDCS [53].

The average stimulation time across all sessions was 13.6 minutes (SEM = 0.59). Stimulation time was not correlated with the magnitude of the anodal tDCS-induced effects on surround suppression r = −0.18, p = 0.6 which suggests that the duration of stimulation did not predict the effect of tDCS on task performance. In addition, a one way ANOVA revealed that there was no sequence effect for any of the conditions (p>0.05).

It has been shown that the change in cortical excitability induced by transcranial stimulation of the primary visual cortex can temporarily modulate contrast sensitivity [37], [38], [54], [55]. In order to ensure that the surround suppression effects could not be explained by a transient change in contrast sensitivity, the contrast threshold of the target alone (control condition) was compared across the behavioural and tDCS sessions (figure 4C). An ANOVA with a factor of stimulation (baseline, anodal and cathodal) indicated that tDCS did not reliably affect the target detection threshold for this particular stimulus (F_2, 18_ = 1.909, p = 0.177) which had a spatial frequency close to the peak of the human spatial contrast sensitivity function [56], and may constitute a ceiling effect for sensitivity improvements [38]. An inspection of figure 4C, however, does suggest that anodal stimulation may have slightly improved contrast sensitivity for the target alone, albeit non-significantly. To investigate whether this played a role in the tDCS induced change in suppression factor, an ANCOVA with a factor of stimulation (baseline, anodal stimulation, cathodal stimulation) and a covariate of anodal tDCS-induced change in target stimulus contrast threshold was conducted. The effect of stimulation on surround suppression was significant even after controlling for any anodal tDCS-induced effects on contrast sensitivity (F_2, 16_ = 5.623, p = 0.014).

## Discussion

Anodal tDCS delivered over the occipital pole reduced surround suppression to the extent that, on average, the presence of a surround mask no longer had any measureable effect on contrast detection threshold (mean suppression factor of 1). This effect was highly specific as anodal tDCS had no effect on overlay suppression and cathodal stimulation had no reliable effect on either overlay or surround suppression. It is our contention that a GABA-mediated effect is the most likely mechanism underlying these findings. Surround suppression has been shown to be significantly reduced in patients with schizophrenia [26] who are thought to have reduced levels of GABA [57]. Moreover, a strong positive correlation has been reported between the strength of surround suppression and GABA concentration within the visual cortex [25]. Similar results have been found in animal models. Fu et al. [27] compared the strength of surround suppression between old and young rhesus monkeys and found a significant reduction of surround suppression in older monkeys. Although GABA concentration was not directly assessed in these animals, it was proposed that the reduced strength of surround suppression could have resulted from an age-related decrease of GABA levels within the visual cortex [58]. In agreement with the present results, the effect of modulating GABAergic inhibition in the visual cortex may be specific to surround suppression, as inactivation of GABAergic inhibitory mechanisms in the primary visual cortex of cats has no effect on overlay masking [32].

These results are consistent with recent work demonstrating that anodal but not cathodal tDCS can reduce GABA mediated inhibition within the human motor cortex [6], [7], [8], [16] and are the first to demonstrate that this effect may also apply to the visual cortex. Based on the link between anodal tDCS induced reductions in GABA concentration in the motor cortex and learning of a motor task [7], these findings provide support for the potential use of tDCS to modulate plasticity within the visual cortex, both as an investigative tool and for purposes of rehabilitation [59].

The data also provide insights into the functional organization of the human visual system by supporting the notion that surround and overlay masking are driven by different neural mechanisms and rely on processes originating from distinct neural loci [28], [29], . Early neurophysiological studies showed that the properties of overlay and surround suppression differ. While surround masking is strongly tuned for orientation and spatial frequency indicating a cortical origin, overlay suppression has little or no tuning in these domains [17], [19]. Consistent with these differences in tuning properties, later work with anesthetised cats indicated that overlay masking may be subcortical in origin, involving processing within the LGN as well as feed-forward inputs from the LGN to V1 [28], [29], [30], [31].

Studies in humans have supported these neurophysiological findings. For example, Petrov et al. [21] provided psychophysical evidence that overlay masking precedes surround masking in visual processing in accordance with the available neurophysiological data [61]. In addition, evidence for surround suppression having a cortical origin in humans has been found using magnetoencephalography [60]. The presence of a surround mask resulted in a decrease in the amplitude of the visually evoked magnetic response but did not affect the latency of the signal. Ohtani et al. [60] proposed that if surround suppression occurred within the retino-geniculate pathway it would have resulted in a retardation of the signal. Based on the absence of a delay in the cortical response to targets with a surround mask, Ohtani et al. concluded that surround suppression may take place in V1 and perhaps V2.

Cathodal tDCS was used as an active control in this study, however it is important to note that cathodal tDCS has been shown to exert an effect on visual brain areas. For example, cathodal tDCS of the primary visual cortex can increase phosphene thresholds [3] and increase [36] or decrease [35] specific components of the VEP. Cathodal tDCS of V1 has also been reported to reduce contrast sensitivity for sinusoidal gratings with a spatial frequency close to the peak of the human contrast sensitivity function [38], but does not seem to influence contrast sensitivity measurements made using threshold perimetry [37]. In addition, cathodal tDCS of V5, a motion sensitive area of the extrastriate cortex, has been reported to improve visuomotor coordination and either improve or impair motion perception depending on the type of motion stimulus used [62]. Based on the current literature, however, cathodal tDCS does not appear to reduce GABA-mediated inhibitory interactions within the cortex such as those putatively targeted by the psychophysical stimuli used in the current study. For example, the administration of the GABA A receptor agonist Lorazapam has no effect on the reduction of excitability within the motor cortex induced by cathodal tDCS [16]. In contrast, Lorazapam completely blocks the ability of anodal tDCS to reduce intracortical inhibition within the motor cortex and leads to a complex change in the after-effects of anodal tDCS on neural excitability that appears to be independent of intracortical inhibition [16]. The present finding that cathodal tDCS did not influence surround suppression is in agreement with this earlier work.

Within the baseline psychophysical data (figure 3), surround suppression was orientation tuned with the strongest suppression occurring when the mask was at the same orientation as the target. This is in accordance with previous psychophysical results in humans [21], [39] and animal neurophysiology [17], [19]. An unexpected finding was the facilitation found for surround mask orientations of 25° and 35°. Comparable psychophysical measurements of surround suppression have been made at 6° of eccentricity and in the fovea (where surround suppression was absent) [21], [39]. However, psychophysical data from [63] demonstrate that the presence of a surround mask can significantly facilitate the detection of a target presented at the fovea if the surround differs in orientation by more than 20° from the target. Yu et al [63] suggest that the presence of the cross oriented surround acts to enhance the signal to noise ratio within the stimulus. It would appear, therefore, that both facilitatory and suppressive mechanisms are present at the eccentricity of 1.2° tested in the present study. Specifically, suppressive mechanisms similar to those found in the periphery are activated when the difference between the surround mask and target orientation is small, whereas facilitatory mechanisms similar to those found at the fovea are activated when the mask and target orientations differ by more than 20°.

The results also show the presence of coarse orientation tuning for overlay masking. This finding does not agree with the neurophysiological data of DeAngelis [17], [19] but is relatively consistent with the psychophysical results of Petrov et al. [21]. Our findings do differ from previous reports in that overlay masks with the same or similar orientations to the target (0° and 5°) resulted in facilitation. This can be explained by the fact that in this study the mask and target had the same spatial frequency and phase. Therefore when mask and target were combined, the contrast of the two stimuli was summed to enable discrimination of the target and mask combination from the mask alone based on a just noticeable difference in suprathreshold contrast (figure 1C). In other words, when the mask orientation was the same or very similar to the target orientation, participants were able to adopt a different strategy for task performance which relied on within channel contrast summation and therefore did not reflect overlay suppression. A clear facilitation of neural responses for overlay mask orientations similar to the target orientation has also been shown neurophysiologically [19], [20], [64].

It has been demonstrated that GABAergic circuitry plays an important role in orientation tuning within the cat [65] and human visual cortex [66]. It would therefore be interesting to evaluate the effects of tDCS on the orientation functions of both types of suppression.

To the best of our knowledge, this study provides the first evidence to suggest that anodal tDCS can reduce intracortical inhibition in the visual cortex adding to the growing body of evidence that anodal tDCS can reduce GABAergic inhibition within the human neocortex. This has important implications for the future therapeutic use of tDCS. For example, GABAergic inhibitory interactions have been implicated in the visual loss that occurs in amblyopia [13], [14], [15], [67], [68] which suggests that anodal tDCS may represent a potential tool in the treatment of amblyopia.
